# Developmental Programming-Aging Interactions Have Sex-Specific and Developmental Stage of Exposure Outcomes on Life Course Circulating Corticosterone and Dehydroepiandrosterone (DHEA) Concentrations in Rats Exposed to Maternal Protein-Restricted Diets

**DOI:** 10.3390/nu15051239

**Published:** 2023-03-01

**Authors:** Elena Zambrano, Luis A. Reyes-Castro, Guadalupe L. Rodríguez-González, Roberto Chavira, Consuelo Lomas-Soria, Kenneth G. Gerow, Peter W. Nathanielsz

**Affiliations:** 1 Departamento de Biología de la Reproducción, Instituto Nacional de Ciencias Médicas y Nutrición Salvador Zubirán, Mexico City 14080, Mexico; 2 CONACyT-Cátedras, Departamento de Biología de la Reproducción, Instituto Nacional de Ciencias Médicas y Nutrición SZ, Mexico City 14080, Mexico; 3 Department of Statistics, University of Wyoming, Laramie, WY 82071, USA; 4 Wyoming Center for Pregnancy and Life Course Health Research, Department of Animal Science, University of Wyoming, Laramie, WY 82071, USA

**Keywords:** aging, life course steroid programming, maternal low protein diet, sexual dimorphism differences, aging serum steroid fall, DHEA, corticosterone

## Abstract

The steroids corticosterone and dehydroepiandrosterone (DHEA) perform multiple life course functions. Rodent life-course circulating corticosterone and DHEA trajectories are unknown. We studied life course basal corticosterone and DHEA in offspring of rats fed protein-restricted (10% protein, R) or control (20% protein, C), pregnancy diet first letter, and/or lactation second letter, producing four offspring groups—CC, RR, CR, and RC. We hypothesize that 1. maternal diet programs are sexually dimorphic, offspring life course steroid concentrations, and 2. an aging-related steroid will fall. Both changes differ with the plastic developmental period offspring experienced R, fetal life or postnatally, pre-weaning. Corticosterone was measured by radioimmunoassay and DHEA by ELISA. Steroid trajectories were evaluated by quadratic analysis. Female corticosterone was higher than male in all groups. Male and female corticosterone were highest in RR, peaked at 450 days, and fell thereafter. DHEA declined with aging in all-male groups. DHEA: corticosterone fell in three male groups but increased in all-female groups with age. In conclusion, life course and sexually dimorphic steroid developmental programming-aging interactions may explain differences in steroid studies at different life stages and between colonies experiencing different early-life programming. These data support our hypotheses of sex and programming influences and aging-related fall in rat life course serum steroids. Life course studies should address developmental programming-aging interactions.

## 1. Introduction

The rodent serum steroids corticosterone and dehydroepiandrosterone (DHEA) regulate multiple key age-related cellular mechanisms across the life-course, such as metabolic function [1] and oxidative stress (OS) [2]. It is, therefore, important to establish data on the trajectory of serum concentrations of both steroids across as much of the life-course as possible. In primates, cortisol is produced in the adrenal zona fasciculata and DHEA in the zona reticularis [3] and, to a lesser extent, gonads [4]. In rodents, corticosterone is secreted by the adrenal cortex while DHEA is locally produced in different tissues (gonads and the nervous system) [5]. Very few normative life course serum steroid concentration studies covering nearly all the life course are available for glucocorticoids and DHEA [6,7,8,9]. An early life course, sexually dimorphic increase and subsequent aging-related serum corticosterone and DHEA fall occur in rat offspring of normally fed and high-fat, high-energy fed mothers [6]. Simultaneous studies of both sexes are limited. Due to the well-recognized sexual dimorphism of the hypothalamic-pituitary-adrenal axis (HPAA) function, it is necessary to compare life course data in both sexes. A major deficiency in most published studies is the lack of longitudinal data from multiple life course time points that permit regression analysis across the whole lifespan rather than categorical analyses from limited time points.

Both high circulating glucocorticoid concentrations in Cushing’s syndrome [10] and low circulating concentrations in Addison’s disease [11] result in premature aging [1]. There is some evidence that DHEA increases longevity and improves important age-related functions such as cognition [12,13]. DHEA is a potential mediator of reactive oxygen species scavenger synthesis and has also been reported to augment insulin sensitivity and peroxisome proliferator activation [14] actions which potentially would reduce OS and lengthen lifespan.

In the present study, rat mothers selected randomly were fed a restricted protein diet (10% protein diet; R) while controls ate 20% protein (C). Control and R mothers were fed from conception through pregnancy and/or lactation, producing four offspring (F1) groups, the first letter maternal diet in pregnancy and the second letter maternal diet in lactation—CC, RR, CR, and RC. All F1 were fed normal laboratory chow diet after weaning. Therefore, differences in outcomes were due to the feeding regimens during these two plastic stages of development. We studied life course circulating corticosterone and DHEA in F1 at six stages of the life course from postnatal day (PND) 21–850 (normal lifespan ~1000 days). Several investigators have studied F1 developmental programming outcomes and functional aging trajectories resulting from similar maternal protein-restricted diets [15,16,17,18]. Despite the fact that programming by a low maternal protein diet has been extensively studied in multiple systems [17,19,20,21,22,23,24,25,26,27], there are no detailed data on life course programming of F1 corticosterone and DHEA by this extensively studied maternal dietary programming challenge in pregnancy and/or lactation. Although maternal nutritional programming research currently focuses more on maternal obesity and high-calorie diets, there are still many areas of the world where the maternal nutritional challenge is a low intake of nutrients, including protein. We aimed to determine life course F1 serum corticosterone, DHEA and DHEA:corticosterone ratio (DHEA:CORT) changes.

This study had two critical and previously little-addressed goals. We sought corticosterone and DHEA life course serum values at six ages spread across approximately 85% of the life course in F1 of these four maternal F1 groups. Maternal low-protein diets have been shown to program F1 metabolism, cardiovascular function and reproductive outcomes [28,29,30,31]. We posed two hypotheses: first, that F1 exposed during development to a maternal low protein diet show sexually dimorphic age-related programming of life course circulating steroid concentrations: second, we hypothesized this F1 programming is dependent on the precise plastic developmental period F1 are exposed to R—fetal life, during lactation or both periods.

## 2. Methods

### 2.1. Animal Care and Maintenance

**Figure 1:**
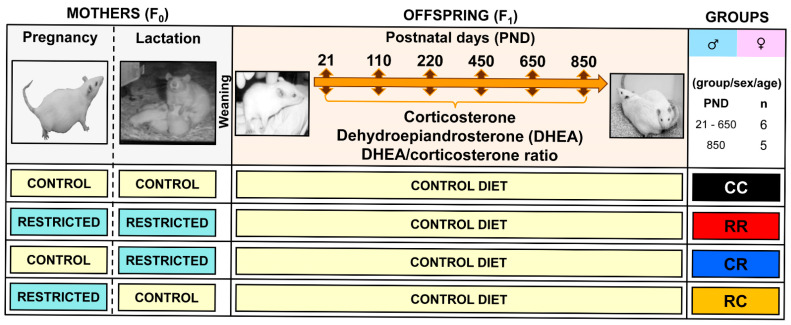
Timeline. Maternal protein restriction design. Female (F0) rats were fed with control (20% protein, C) or protein restricted (10% protein, R) diet during pregnancy and/or lactation, producing four offspring groups; first letter pregnancy, second lactation diet—CC, RR, CR, and RC. After weaning, both male and female offspring were fed with the control diet until postnatal day (PND) 850. Serum samples were taken at PND 21, 110, 220, 450, 650 and 850. n = 6 per group/sex/age with the exception of PND 850, in which n = 5.

Diet and breeding details are published [32]. Briefly, Wistar rats ate Purina Laboratory Chow 5001. Lighting was on from 07:00 to 19:00 h. All procedures were approved by the Instituto Nacional de Ciencias Medicas y Nutricion, Salvador Zubiran, Mexico City Animal Experimentation Ethics Committee (INCMNSZ, BRE-105). We studied a rat model of offspring programming by maternal protein-restricted diet (R—10% protein). Female rats were bred around postnatal day (PND) 120. Prior to pregnancy, all breeding females were fed a control diet (C—20% protein). C and R diets were fed in either pregnancy and/or lactation to produce offspring of four maternal groups—first letter pregnancy diet, second letter lactation diet—CC, RR, CR and RC. Food and water were available ad libitum. Delivery day was considered PND 0. To ensure offspring homogeneity, litters with less than 10 or more than 14 pups were excluded from the study. Litters of 12–14 pups were adjusted to 12, and litters of 11 pups were retained as 11 while maintaining as close to a 1:1 sex ratio as possible. After weaning (PND 21), all offspring ate a C diet. Blood was obtained from one offspring in the litter on PND 21, 110, 220, 450, 650, and 850 (Figure 1). One rat was bled separately at each age. The subjects were mostly siblings. In our colony, the normal life span is ~1000 days in the offspring of control-fed mothers [32].

### 2.2. Steroid Measurements

After 6 h of fasting, between 12:00 p.m. and 2.00 p.m., rats were anesthetized with isoflurane and decapitated [33]. For each group, trunk blood was collected, centrifuged at 2880 RCF for 15 min at 4 °C to remove red blood cells, and serum was frozen until assayed. Serum corticosterone concentrations were determined using a commercial rat DPC Coat-a-count kit (TKRC1) (Diagnostic Products, Los Angeles, CA, USA) [33] and DHEA serum concentrations were determined by enzyme-linked immunosorbent assay (ELISA) using a commercial kit DRG Instruments GmbH (Marburg, Germany, cat #: EIA-3415) [34]. Other publications [35,36] have used ELISA kits to measure DHEA and found concentrations similar to ours [33].

### 2.3. Statistical Analysis

We conducted a quadratic analysis of life course changes in all endocrine variables. PND 21 values were excluded from analysis in all data sets due to proximity to weaning with its attendant marked influence on the offspring of lactation and approach of puberty, which is accompanied by many rapid changes occurring over just a few days. Corticosterone values at PND 110 and 220 were similar and also similar at PND 650 and 850. With this in mind, we simplified the construction of the life course corticosterone trajectory by restricting the corticosterone data analysis to PND 220, 450, and 650. From these trajectories, we calculated the timing of the peak corticosterone concentration and its value. PND 21 DHEA was excluded for similar reasons to corticosterone values. PND 850 DHEA values were included in the quadratic analysis since there was a continued DHEA fall in RR in both sexes and RC in males.

We analyzed the two sexes separately. CC female corticosterone was higher than male at PND 220 and 450 (*p* < 0.01 by non-paired t-test), CC female DHEA was higher than male at all ages except PND 220 (*p* < 0.05), and DHEA: CORT ratio lower at PND 220 and 450 (*p* < 0.01). Therefore, the sexes were analyzed separately throughout.

## 3. Results

### 3.1. Effect of Timing of Maternal Dietary Challenge on Aging Trajectory of Offspring Serum Corticosterone

**Figure 2:**
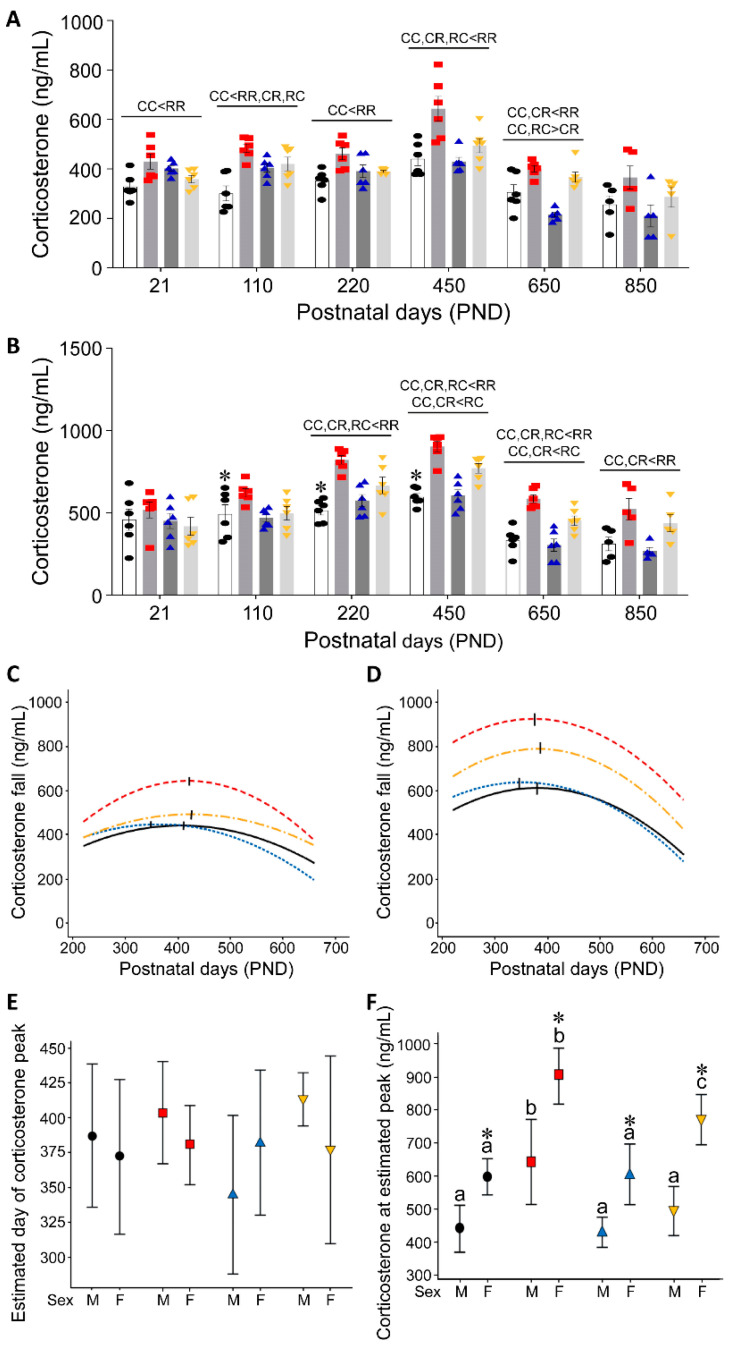
Serum corticosterone in (**A**) male and (**B**) female: histograms averaged data, individual animal data: CC (open histograms, black circles), RR (grey histogram, red squares), CR (grey histogram, blue triangles) and RC (light-grey histogram, yellow inverted triangles) at six life course stages. Data are mean ± SEM, all groups n = 6 except PND 850, n = 5. Differences between the groups of the same sex are indicated on the lines above each age (*p* < 0.05). * *p* < 0.05 male vs. female in C groups at the same age. (**C**) male and (**D**) female show best fit quadratic curves for the corticosterone fall in each group: CC solid black, RR red dash, CR blue dash and RC yellow dash line (PND 220, 450 and 650). (**E**). The male and female estimated days of corticosterone peaks were similar in all groups. (**F**). Female corticosterone concentrations were higher than males in all groups (* *p* < 0.05).

Individual and group male and female corticosterone serum concentrations across the life course are shown in Figure 2A and Figure 2B, respectively. Significantly different groups are indicated above lines at each age. Male (Figure 2C) and female (Figure 2D) best fit quadratic curves show the timing of peak values with a vertical line. Day of peak values was similar in all groups (Figure 2E). At the estimated peak, female corticosterone was greater than male in all groups (Figure 2F). RR corticosterone was greater than all other groups in both sexes. RC females were greater than CC and CR females (Figure 2F).

### 3.2. Effect of Timing of Maternal Dietary Challenge on Aging Trajectory of Offspring Serum DHEA

**Figure 3:**
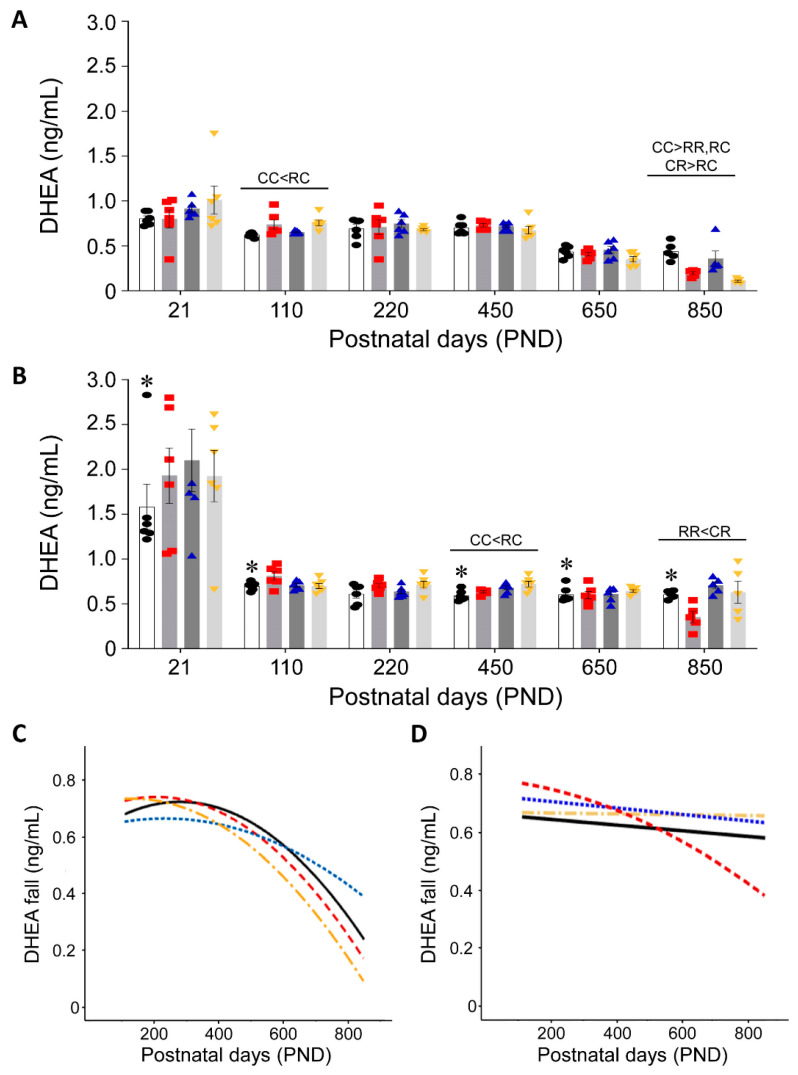
Serum DHEA in (**A**) male and (**B**) female: CC (open histograms, black circles), RR (grey histogram, red squares), CR (grey histogram, blue triangles) and RC (light-grey histogram, yellow inverted triangles) Data are mean ± SEM, group n = 6 per group at all ages except PND 850 where n = 5. Differences between the groups of the same sex are indicated on the lines above each age (*p* < 0.05). * *p* < 0.05 male vs. female in C groups at the same age. (**C**) male and (**D**) female show best-fit curves for the DHEA fall in each group: CC solid black, RR red dash, CR blue dash and RC yellow dash line.

Individual and group male and female DHEA serum concentrations are shown in Figure 3A and Figure 3B, respectively, with significantly different groups indicated above lines at each age. Best-fit quadratic curves are presented in males (Figure 3C) and females (Figure 3D). Figure 3C, D show that serum DHEA concentrations are similar in both sexes until about PND 400. Thereafter, male serum DHEA concentration falls rapidly in RR, RC, and CR, and slightly slower in CC. A quadratic is a superior fit in all cases. In females (Figure 3D), there is a little trend for the three groups and a modest (quadratic) decline in RR.

### 3.3. Effect of Timing of Maternal Dietary Challenge on Aging Trajectory of Offspring Serum DHEA:CORT

**Figure 4:**
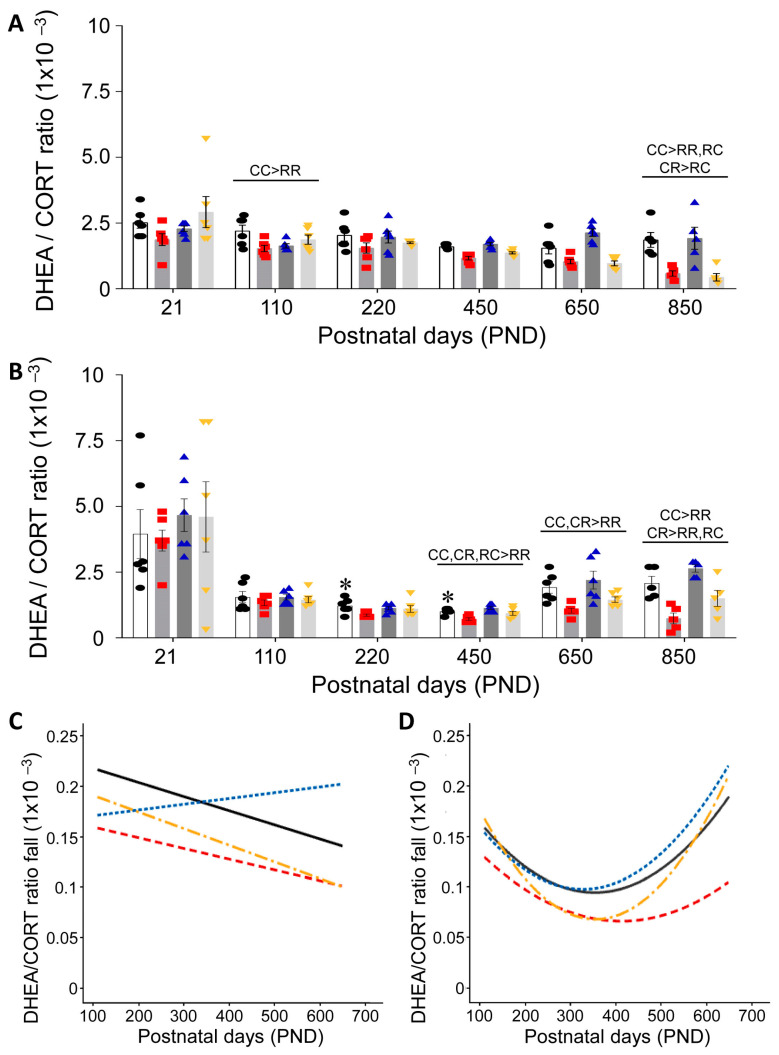
Serum DHEA:CORT ratios are presented in (**A**) male and (**B**) female: CC (open histograms, black circles), RR (grey histogram, red squares), CR (grey histogram, blue triangles) and RC (light-grey histogram, yellow inverted triangles) at six life course stages. Data are mean ± SEM, group n = 6 at all ages except PND 850 where n = 5. Differences between the groups of the same sex are indicated on the lines above each age (*p* < 0.05). * *p* < 0.05 male vs. female in C groups at the same age. (**C**) male and (**D**) female show best-fit curves for the DHEA:CORT ratio fall in each group: CC solid black, RR red dash, CR blue dash and RC yellow dash line.

Since DHEA has been considered by many investigators as extending lifespan, we evaluated the individual and group male and female DHEA:CORT ratio values (Figure 4A,B, respectively). Significantly different groups are indicated on the lines above the ages.

Life course serum DHEA:CORT ratio trajectories show marked sexual dimorphism. In males (Figure 4C), the trajectories are linear, rising slightly in CR but falling in the other three male groups. Female ratios (Figure 4D) are similar to males but show concave-up curves that increase in all-female groups in the second half of life.

### 3.4. Effect of Timing of Maternal Dietary Challenge on Daily Mean Corticosterone, DHEA and DHEA:CORT Ratio

**Figure 5:**
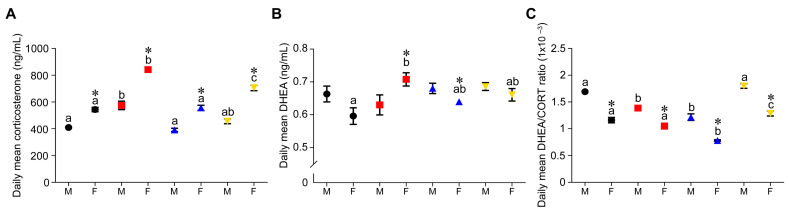
Mean daily values. (**A**) Serum corticosterone, (**B**) Serum DHEA and (**C**) Serum DHEA:CORT ratio in male (M) and female (F) in the four groups: CC (black circles), RR (red squares), CR (blue triangles) and RC (yellow inverted triangles). Data are mean ± SEM, group n = 6 per group at all ages except PND 850 where n = 5. *p* < 0.05 for data not sharing at least one letter within different groups of the same sex. * *p* < 0.05 male vs. female.

Female daily mean corticosterone was higher than male in all groups (*p* < 0.05). RR corticosterone serum levels were higher than the other groups in males and females, and RC was higher than CC and RC in females (Figure 5A). There were no daily mean DHEA differences in male groups, but RR was higher than CC in females (Figure 5B). RR and CR females had higher and lower daily mean DHEA than males, respectively (*p* < 0.05) (Figure 5B). Female daily serum DHEA:CORT ratio was lower than males in all groups (*p* < 0.05) (Figure 5C). CR and RC were lower and higher than CC and RR, respectively (*p* < 0.05) (Figure 5C).

## 4. Discussion

### 4.1. Need for Studies That Address Life-Course Programming of Circulating Glucocorticoids

Since glucocorticoids play multiple key metabolic roles at different stages of the life course, the first step in understanding the role of glucocorticoids in aging is to establish the precise timing of life course changes in basal circulating glucocorticoids in relation to normal aging. We identified one of the major obstacles to filling this need is the nature of the available published data. In most age-related glucocorticoid studies, data are only presented at a few ages—generally two or, at the most, three [6,16]. In addition, well-accepted markers of aging and frailty, such as grip strength, are changing as early as one-third of the way through the lifespan [37]. Therefore, to understand the antecedents of aging, steroid values are needed well before any of the known hallmarks of aging emerge [38]. In addition, it is essential that robust life course data contains values from as many ages across the lifespan as practically possible. Studies in both sexes are required as we have shown marked life course and sex-specific changes in serum corticosterone in the rat [6]. A recent life-course study on the trajectory of mortality index with age shows marked differences between the two sexes. Importantly in relation to male and female differences, the relationship between males and females is constantly changing [39].

### 4.2. Criteria for Studies That Address Life-Course Programming of Circulating Glucocorticoids

As mentioned above, to characterize life course aging changes in circulating glucocorticoids, blood sampling should begin as early in life as possible in order to address the potential early emergence of developmental programming outcomes. The plastic periods in which developmental programming can occur and lead to pathologies such as diabetes and vascular and endocrine comorbidities that influence the study subject’s lifespan are even seen in fetal life [40,41,42,43]. In order to standardize aging confounds, we started data collection at weaning in our well-established, in-house colony of rats that have homogeneous developmental histories and known maternal and paternal phenotypes [32]. It is important to note that this vital developmental information is usually unavailable in commercially sourced animals. Studies need to ensure homogeneity and lack of cofounds, such as siblings within a study group. Siblings within a group biases data due to the excessive influence of programming by a single or small group of mothers.

There is much debate about the life course of glucocorticoids and DHEA changes. We have now studied life course changes in circulating corticosterone and DHEA in twelve independent groups of rats, the eight groups presented here and four groups in a study of offspring of obese mothers [6]. All groups showed a similarly timed age-related fall in serum corticosterone, though with different absolute group blood concentrations. Since we conducted these studies with homogeneous animals of uniform backgrounds, we hope these findings provide firm information for this important debate, and it is clear that corticosterone concentrations fall in the second half of the rat life course. Clearly, there are differences in the developing HPAA response to the same maternal nutritional programming challenge presented in different developmental windows. For example, since the steroid response to RR is greater than RC in both sexes, it would appear that programming influences are present in both pregnancy and lactation. The similarities in the timing of the corticosterone peaks in all groups would suggest HPAA neural regulation. It is of interest that two independent studies in a nonhuman primate, the baboon, show a fall in cortisol across the life course with remarkable similarity in the rates of fall coefficient [7,9]. In baboons, DHEA falls similarly across the life course in males and females [8].

### 4.3. Chronobiology of the HPAA System and Importance of Blood Sampling Time 

The HPAA is subject to multiple internal and external environmental influences that change markedly across the life course, such as stress and nutrition. All published studies addressing basal HPAA physiology, including our own, inevitably include the effects of confounds produced by the study design. In light of the well-known circadian rhythms that affect the HPAA, sampling frequency and timing will affect findings. Our samples were obtained 5–7 h into the light period, the resting time of rodent sleep-wakefulness when metabolism is basal and stable. Another study in 60-day-old male rats showed similar early light-phase corticosterone low baseline stability [44]. We sampled in the final two hours of this period of greatest corticosterone stability. At other times, serum corticosterone may change by 50% within a 2-hr period [44].

Indwelling catheters and tethers have been used to allow frequent, relatively unrestrained sampling of rhythms and rapid variations. However, these approaches also introduce the effects of surgical instrumentation and mobility restriction. The characteristics of HPAA rhythmicity have been extensively reported in several nonhuman primate studies [45,46,47]. DHEAS is observed to decline with age. A 24-h rhythm study of steroid values at 10 and 26 years showed that maximum, mean and minimum cortisol values were increased in the older group, especially in the light, the active phase of the day [46].

Multiple functions of DHEA have been described [48]. For example, it has been implicated in reproductive functions since it can be bioconverted into estrogens and testosterone [49]. It is also involved in stress regulation due to its anti-glucocorticoid activity [50]. DHEA counteracts a variety of negative effects of excessive glucocorticoid action, e.g., on visceral obesity and decreased insulin sensitivity in elderly individuals [51]. During acute stress, DHEA concentrations are increased, but in chronic stress, the DHEA response is attenuated. Also, DHEA promotes neuroplasticity, neurogenesis and neuroprotection [52]. Since many of the functions of DHEA counteract or act opposite to those of cortisol, it is important to study their actions together. One of the alternatives is not only to express the concentration of each of these hormones but also to indicate the ratio [53]. Since DHEA is protective against aging, the maintenance of the female ratio in later life in comparison with the male fall in the DHEA:CORT ratio may represent one of the mechanisms responsible for the so-called “female aging advantage” [39]. It is clear that there is marked sexual dimorphism in these later life changes.

### 4.4. Potential Mechanisms Responsible for the Fall in Corticosterone in the Second Half of Life

The absolute level of circulating corticosterone is mainly determined by the setting of the activity level of the HPAA. In vitro studies of steroid production by dispersed adrenal cells from 2, 5, 12, and 18-month-old rats show decreased corticosterone production and adrenocorticotropic hormone (ACTH) responsiveness as rats age [54]. With age, fresh male rat adrenal homogenates become less able to synthesize cholesterol for steroidogenesis [54]. 3-hydroxy-3-methylglutaryl coenzyme A (HMG-CoA) activity is lowest at PND 365 and 550. Interestingly, this decrease is not associated with decreased activity of steroidogenic enzymes [54]. Male rat corticosterone production by isolated adrenal cells in response to ACTH and cyclic adenosine monophosphate (cAMP) has been studied from 6–24 months. Aged adrenocortical cells lose most of their corticosterone production in response to both ACTH and cAMP. The authors state, “Analysis of the data suggests that from 6 to 12 months, an intracellular steroidogenic lesion develops; in addition, there may be a loss in ACTH receptors on the plasma membrane. After 12 months, these defects increase and are accompanied by a decrease in receptor sensitivity to ACTH”. These data support our findings of a fall in circulating corticosterone that begins around mid-life [55].

Changes in cellular miRNAs are another potential mechanism responsible for age-related decreases in glucocorticoid production. ACTH and dexamethasone control several miRNAs in adrenal steroidogenic cells [56]. These findings further increase the number of mechanisms that merit investigation in order to determine the mechanisms responsible for the fall in glucocorticoids across the life course demonstrated in this study. Since steroids are products of the mitochondria, studies are needed on the changes in relevant mitochondrial function in zona fasciculata with age.

### 4.5. Mechanism Responsible for Different Corticosterone Concentrations in the Different Programming Outcomes

Maternal rat corticosterone is increased as a result of protein restriction at 19 days gestation. This increase results in altered offspring HPAA function postnatally when corticosterone is lower in both male and female RR pups at PND 2 than control, presumably because of increased negative feedback by the high maternal concentrations a few days previously [15,57].

The setting of the HPAA in rats has been shown to be modified by developmental programming. Mothers of different strains of Long Evans rats show different amounts of licking and grooming of their pups during lactation. The amount of maternal licking and grooming over the first week of the pup’s newborn life strongly influences the pup’s later-life response to stress. Offspring of mothers who perform low amounts of licking and grooming have fewer glucocorticoid receptors in their hypothalamus, resulting in lower negative glucocorticoid feedback on the HPAA. In contrast, high levels of maternal care, licking and grooming programs more hypothalamic glucocorticoid receptors. As a result, when stressed, offspring exposed to a greater amount of maternal care secrete less ACTH and glucocorticoids than offspring of low-licking and grooming mothers [58]. Sexual dimorphism has been shown in the programming of HPAA feedback. In one study, female, but not male, offspring of mothers stressed by environmental changes during pregnancy showed increased HPAA [59]. Alteration of hypothalamic glucocorticoid receptors following programming by different maternal nutritional challenges might result in decreased negative glucocorticoid HPAA feedback and resultant increased corticosterone secretion.

In keeping with the view that the level of HPAA negative feedback is altered by maternal protein diet programming, male rat corticosterone concentrations were higher in RR than CC offspring before, during and after 20 min immobilization [60]. Similar changes were observed in females, although the response to the stress was lower [61]. The cellular mediators of these effects of maternal separation remain to be established. There are many examples of the effects of challenges during development on the later life function of the HPAA. In one rat study, maternal separation at PND 10 increased pup adrenal expression of steroidogenic acute regulatory protein and steroidogenic enzymes [62].

### 4.6. Differences in Corticosterone and DHEA Life-Course Trajectories and Metabolic Consequences for the Health Span

In primates, cortisol is produced in the adrenal zona fasciculata and DHEA in the zona reticularis [3] and, to a lesser extent, gonads [4]. DHEA is a precursor in the production of testosterone and estrogen, but its androgenic effect is weak [4]. Serum DHEA concentrations are relatively high in the fetus and neonate, low during childhood and increase during puberty [63]. In rodents, corticosterone is secreted by the adrenal cortex while DHEA is locally produced in different tissues (gonads and the nervous system). Gonads contribute to circulating DHEA, and its synthesis may be regulated by factors independent from those involved in the stress response, which should be carefully considered when analyzing DHEA to study HPAA activation, as gonadal or placental DHEA production may bias the putative adrenal response. In rats, the highest levels of DHEA were observed in the spinal cord compared to plasma. The pathway of DHEA synthesis in the rodent brain is controversial, as the 17α-hydroxylase (CYP17A) enzyme expression is low in adult rats, and alternative CYP17A-independent pathways were suggested. In several rat, bovine, and human brain model systems, DHEA biosynthesis is mediated by oxidative stress/Fe^2+^, independent of the CYP17A enzyme. This mechanism is not fully understood [5]. Corticosterone and DHEA functions and metabolism, although somewhat interactive, are different. Therefore, it is not surprising that the trajectories of the two steroids are different.

One key aging review describes nine hallmarks of aging: genomic instability, telomere attrition, epigenetic alterations, loss of proteostasis, de-regulated nutrient sensing, mitochondrial dysfunction, cellular senescence, stem cell exhaustion, and altered intercellular communication [38]. All of these hallmarks need investigation in relation to the life course of the steroids corticosterone and DHEA, whose changes we describe here. To the best of our knowledge, no specific studies have been designed to specifically address the effects of glucocorticoids on the nine markers of aging. In the fetal primate liver, proteasome and oxidative phosphorylation genes are down and miRNA changes in fetuses of obese mothers [64].

DHEA decreases with age in men and women [65]. DHEA is a weak estrogenic precursor and may play a role in the female aging advantage [39]. The nature of the role DHEA plays in aging is controversial but has been suggested to be biphasic, with protection from aging at high concentrations and increased susceptibility at low concentrations [66]. Glucocorticoids and glucocorticoid receptors are found in mitochondria, and treatment with glucocorticoids alters mitochondrial DNA transcription. Thus, decreased glucocorticoid and glucocorticoid receptors could play a role in decreased mitochondrial steroid production [67].

The caloric restriction, which extends the life span and improves age-related disease, is also associated with a decreased fall in DHEA [68]. Glucocorticoids have both pro and apoptotic effects [69], and whether a pro-apoptotic or anti-apoptotic effect is induced is often tissue- and/or cell type-specific [69]. There have only been a few studies of the effects of glucocorticoids on autophagy, another age-related cellular process. Glucocorticoids stimulated the transcription of autophagy genes such as ATG5, LC3, Beclin 1, and SQSMT1/p62, as well as activated AMPK to promote muscle atrophy [70].

Elevation of corticosterone in rodents results in the development of hepatic steatosis [14,71]. We have shown elevated early stages of non-alcoholic fatty liver disease (NAFLD) in the offspring of obese rats fed high-fat diets. It would be valuable to observe changes in the offspring of protein-restricted mothers with similar life-course corticosterone changes.

One of the most valuable studies that should be conducted in the future on the effects of these life course corticosterone changes would be to administer corticosterone to mimic the RR profile in CC rats and observe effects on the nine markers of aging mentioned above.

Limitations of this study. We restricted our studies to rodents and acknowledge that considerable data in nonhuman primates and humans indicate an increase in circulating steroids as well as a decrease (our own data and from others in the baboon). However, as remarked earlier, additional stimuli to the study subjects will undoubtedly affect the corticosterone values. It is clear that species differences, especially in developmental programming and basal metabolic rate, need further investigation. One major limitation is that these data do not test the dynamic aspects, such as steroid secretion rates in response to specific stressors.

Strengths. The strengths of the study are the large portion of the life course ~85% over which corticosterone and DHEA values are obtained, the representation of both sexes to enable the evaluation of sexual dimorphisms and the comparison of the four different programming backgrounds with relatively high n values in both sexes.

## 5. Conclusions and Implications

This comprehensive study addresses three major features of life-course changes in circulating corticosterone and DHEA in rats. (1) First, we studied four different maternal nutritional groups covering pregnancy and lactation together and separately -CC, RR, CR and RC. The data show different effects of the period offspring experienced maternal nutrient restriction, fetal life or postnatally, during lactation pre-weaning. Many studies only report two dietary groups, CC and RC, because they are directed only at the issue of catching up. Male and female corticosterone concentrations were highest in RR, peaking around 450 days and falling thereafter. DHEA concentrations declined with aging in all the male groups but only in the RR female group. (2) By reporting data from both sexes, we determine the extent of sexual dimorphism. Many studies still only report values in one sex, even though sexual dimorphism is a major principle of both programming and aging. Female corticosterone concentrations were higher than males in all groups. DHEA has anti-aging actions, and thus the fall in the aging DHEA:CORT ratio in three male groups compared with an increase in all female groups may relate to the well-documented longer female than male life span. Potential mechanisms for the difference in life course steroid trajectories include the reported differences in the programming of HPAA glucocorticoid receptors and hence negative feedback. (3) Finally, we once again strongly state the fundamental need for data across the whole life course to clarify the current conflicting opinions on the life-course trajectory of circulating hormones such as corticosterone and DHEA.

**Figure 6:**
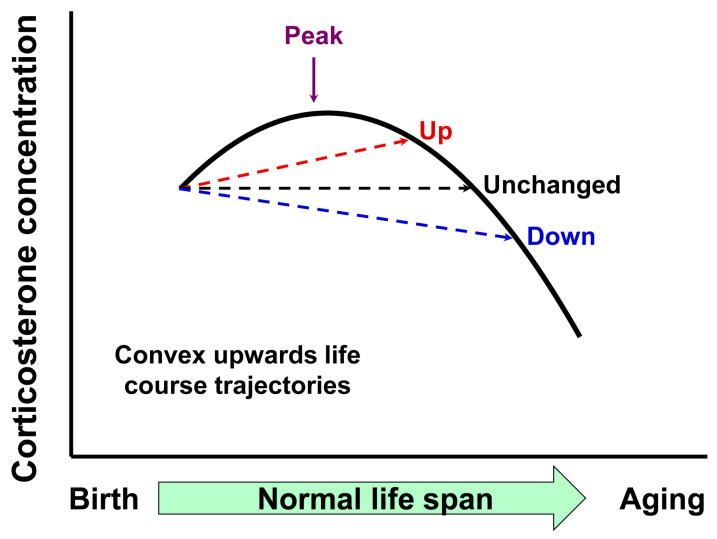
The importance of categorical life course points in aging studies. The figure exemplifies the lifetime corticosterone concentration having a peak at approximately mid-life (purple arrow). If only two samples are obtained, the possibility exists of false conclusions as to whether values rise (red arrow), are unchanged (black arrow) or fall (blue arrow) with age. Hence, data interpretation in the lifespan trajectory depends on the time at which the sample is obtained.

By obtaining concentrations at six ages across the life course, we showed clearly that the trajectory is convex upwards with a peak around halfway through life. When data are limited to a few categorical life course points, often one young and one old, the possibility exists of false conclusions as to whether values rise, are unchanged or fall with age. This possibility is illustrated in Figure 6, where the precise choice of the two categorical time points can lead to the conclusion that the hormone rises with aging, is unchanged across the life course or falls with aging. With multiple data points at different ages, it is possible to determine the situation at either any one point or overall life course trajectory.

Regarding relevance to aging, the data in this manuscript show a clear age-related steroid fall in all eight groups. We [7] and others [9] have shown a fall in cortisol in the second half of nonhuman primate life that shows some similarity to the fall in rat corticosterone reported here, providing a translational link between rodent and human steroid changes with age.

## Data Availability

The data supporting the research for this study is available within the manuscript.
